# Spatially and Temporally Distributed Complexity—A Refreshed Framework for the Study of GRN Evolution

**DOI:** 10.3390/cells11111790

**Published:** 2022-05-30

**Authors:** Alessandro Minelli, Alberto Valero-Gracia

**Affiliations:** 1 Department of Biology, University of Padova, Via U. Bassi 58B, 35132 Padova, Italy; 2 Natural History Museum, University of Oslo, Blindern, P.O. Box 1172, 0318 Oslo, Norway; alberto.valero-gracia@nhm.uio.no

**Keywords:** phenotypic plasticity, polyphenism, polymorphism, adultocentrism, development, hierarchy, unicells, multicellular organisms

## Abstract

Irrespective of the heuristic value of interpretations of developmental processes in terms of gene regulatory networks (GRNs), larger-angle views often suffer from: (i) an inadequate understanding of the relationship between genotype and phenotype; (ii) a predominantly zoocentric vision; and (iii) overconfidence in a putatively hierarchical organization of animal body plans. Here, we constructively criticize these assumptions. First, developmental biology is pervaded by adultocentrism, but development is not necessarily egg to adult. Second, during development, many unicells undergo transcriptomic profile transitions that are comparable to those recorded in pluricellular organisms; thus, their study should not be neglected from the GRN perspective. Third, the putatively hierarchical nature of the animal body is mirrored in the GRN logic, but in relating genotype to phenotype, independent assessments of the dynamics of the regulatory machinery and the animal’s architecture are required, better served by a combinatorial than by a hierarchical approach. The trade-offs between spatial and temporal aspects of regulation, as well as their evolutionary consequences, are also discussed. Multicellularity may derive from a unicell’s sequential phenotypes turned into different but coexisting, spatially arranged cell types. In turn, polyphenism may have been a crucial mechanism involved in the origin of complex life cycles.

## 1. Introduction

In a nutshell, gene regulatory networks (GRNs) are “[s]emi-autonomous regulatory modules responsible for characters or phenotypes” [1] (p. 398). The autonomy of these modules translates into restrictions both in their spatial domains of expression and in the temporal frames of the ontogenetic sequence in which they operate. Following early uses to visualize the context-dependent regulation of enzyme expression in bacteria [2,3], the GRN metaphor has been extensively applied to eukaryotes, especially metazoans [4].

The mostly implicitly assumed background to this spatial and temporal partitioning agrees with the traditional description of individual development (egg to adult), as well as with the hypothesized hierarchical organization of developmental processes and the resulting phenotypes. As in Yen-Chung Chen and Claude Desplan’s definition, “a gene regulatory network is the sum of the interactions between genes in time and space and can be further partitioned into interconnected subnetworks that serve specific functions.” [5] (p. 92).

The analysis of the ontogenetic processes unfolding under the control of GRNs, as outlined by Eric H. Davidson [6], implies a precise explanation of the development and structural organization of multicellular organisms. However, this link between “development theory” and “animal theory”, and the interpretation of development in terms of GRNs, deserves critical examination, as it may pave the way toward a different reading of these processes and their evolution.

By relating genotypic changes with their phenotypic consequences, developmental programs play a central role in defining the boundaries within which selection can drive phenotypic change, thereby playing a profound role in shaping the evolutionary trajectories of species [7] (pp. 1–2).

The above statement, from a recent article entitled “The GRN concept as a guide for evolutionary developmental biology”, encapsulates three main critical aspects of current explanations of developmental processes in terms of GRNs: (i) a reductionist interpretation of the relationship between genotype and phenotype; (ii) a zoocentric vision that ignores the peculiarities of the development and evolution of plants and unicellular organisms; and (iii) an excessive weight attributed to the modularity of the morpho-functional organization of the animal. This modular vision of body organization, and of the regulatory systems that control development, is indeed rooted in two assumptions: (a) an adultocentric view of development and (b) a hierarchical interpretation of body structure to which a similarly hierarchical organization of development must correspond. In this work, we will constructively criticize the aforementioned assumptions.

Our appraisal preserves the very notion of a GRN and the current understanding of its architecture but challenges the correspondence between the overall regulatory scheme and the structural and functional logic of the organism. These aspects are discussed in the following sections:

- Developmental genes?
- Developmental patterns vs. processes.
- Adaptational and teleological views.
- The development of unicells and its regulation.
- Structural and functional hierarchies.
- Spatial and temporal dimensions of development.

## 2. Developmental Genes?

The term “developmental gene” is unquestionable if used for genes of which mutants alter the normal ontogenetic course or for genes whose patterns of expressions are correlated with specific times and events during development. However, we cannot take any of them as directly and uniquely responsible for the origin of an organ or the shaping of the body. We must consider genes in context with the whole cellular environment, not just with other genes [8,9,10]. The network of molecular interactions within any developmental system is so complex and intertwined that a gene can be considered to initiate a sequence of events only if our investigation starts at that point [11]. Nevertheless, the switches between dramatically alternative phenotypes, including femaleness vs. maleness or the presence vs. absence of wings, is often under epigenetic control.

Any discussion of *polyphenism* vs. *polymorphism* requires a preliminary clarification of terminology. Within a species, the occurrence of two or more distinct phenotypic classes, other than developmental stages, is traditionally described as *polymorphism*. Examples of polymorphism include the two sexes in gonochoric species, the different castes in eusocial hymenopterans, the different chromatic forms of the same species of ladybug, or the different specialized forms of polyp and medusa co-occurring in a siphonophore’s colony. In recent decades, however, the term polymorphism has been increasingly restricted only to the instances in which the alternative phenotypes can be attributed to genetic differences. The term *polyphenism* is used instead when development toward one or the other form can be attributed to external influences and, therefore, takes place in the absence of genetic differences (i.e., phenotypic plasticity [12,13,14]). However, precise information on the mechanisms involved is generally lacking [15]; moreover, the divide between polymorphism and polyphenism can be very thin [16]. We should therefore have a flexible approach while collecting classes of phenomena that traditionally would be considered as separate due to the parameters used to describe them.

As a rule, the notion of phenotypic plasticity is not applied to describe differences between subsequent developmental stages. This is despite the valid observation of H. Frederik Nijhout [17] that different life stages of the same organism can be considered as distinct phenotypes of a sequential (ontogenetic) polyphenism. However, there are good reasons to relax the contrast between developmental events along the history of the individual and the differences between equivalent stages of successive generations in multi-generation life cycles [18]. This sounds reasonable in cases of cross-generational plasticity, such as an aphid’s wingless parthenogenetic generation followed by a winged amphigonic generation in the annual cycle of the species. Another textbook example of seasonal polyphenism is the European butterfly *Araschnia levana*, with a spring generation featuring predominantly orange wings with black spots and a summer generation with orange, white, and black bands. The switch between these generations is determined by the photoperiod in the season in which the caterpillar is growing, causing differences in the hormonal control of molting and metamorphosis [19].

All instances of polyphenism mentioned above involve physically separate individuals or different developmental stages of the same individual (in the case of sequential ontogenetic polyphenism). However, polyphenism does not necessarily require physical separation; colonies including two, three, and even more types of zooids are common in some groups of marine invertebrates, such as cnidarians, bryozoans, and doliolid tunicates [20,21].

Polyphenism does not rule out the existence of genetically based individual variation in the response to external cues. Nevertheless, “[w]e are no longer constrained to hypothesize ‘genes for plasticity’ because we are beginning to understand how the different parts of the mechanism that generates the phenotype respond to specific environmental variables. What we observe as phenotypic plasticity is due to the plasticity of a broad diversity of developmental processes that underlie the phenotype.” [22] (p. 589).

## 3. Developmental Patterns vs. Processes

### 3.1. Adultocentrism

The study of biological development is extensively pervaded by adultocentrism [23]. In the case of animals, this means that development is considered as the sequence of changes that turn the egg into a multicellular reproductive stage (the adult). This developmental sequence is accompanied by a progressive increase in topographical, morphological, and functional complexity, although with some exceptions. It thus seems legitimate to attribute the regularity and predictability of such changes to the existence of a regulatory mechanism that controls the different phases and levels of development. In Isabelle S. Peter and Eric H. Davidson’s words:

Development progresses from phase to phase, and this fundamental phenomenon reflects the underlying sequential hierarchy of the GRN control system. In the earliest embryonic phases, the function of the developmental GRN is establishment of specific regulatory states in the spatial domains of the developing organism. In this way, the design of the future body plan is mapped out in regional regulatory landscapes, which differentially endow the potentialities of the future parts. Lower down in the hierarchy, GRN apparatus continues regional regulatory specification on finer scales. Ultimately, precisely confined regulatory states determine how the differentiation and morphogenetic gene batteries at the terminal periphery of the GRN will be deployed.

[24] (p. 970)

However, development is not necessarily the sequence of changes from egg to adult: “We do not witness the birth of a new being: we only see a periodic continuation”; “all morphological change is contained in the previous state”; “there is no morphology without predecessors. Things happen this way because the being is in a way imprisoned in a series of conditions from which it cannot escape, since they are always repeated in the same way internally and externally” [25] (pp. 331–333; our translation). In this perspective, development is “a highly constructive process, where a given aspect of phenotype formation builds upon a pre-existing phenotype created during previous stages of development” [26] (p. 7); see also [27,28,29,30]. In other terms, development goes on, so far as each phase is compatible with the preceding one, whereas the adultocentric view of development requires that each phase is compatible with the following.

If we abandon the adultocentric perspective (and we should), the history of the individual becomes even more interesting: the succession of stages from egg to adult is no longer the development but a particular history of development.

### 3.2. GRN in Blastogenesis

Much of the literature about the regulation of embryonic and post-embryonic development refers to deuterostomes with “type I development”. The term type I development was introduced by Eric H. Davidson for “a general form [of development] characteristic of most invertebrate taxa of today, in which lineage plays an important role in the spatial organization of the early embryo, and cell specification occurs in situ, by both autonomous and conditional mechanisms.” [8] (p. 1). However, within this group, there are species in which development can occur both through normal embryogenesis, followed by a larval stage that will metamorphose into an adult, and by blastogenesis, starting from a multicellular bud and bypassing the embryonic and larval stages [31].

Comparative information about the regulatory systems in place during embryogenesis and blastogenesis is available for the colonial ascidian *Botryllus schlosseri*. This was first explored by studying the expression of individual “developmental genes”. In *Botryllus schlosseri*, *Bs-Pitx*, a homeobox gene involved in the control of left–right asymmetry and organogenesis, is expressed in identical domains during both developmental sequences, but its expression patterns at early development differ deeply [32]. *Wnt* signaling is involved in the establishment of body axes during both embryogenesis and asexual development, suggesting that patterning mechanisms driving morphogenesis are conserved; yet, during regeneration, the determination of body axes occurs independently of tissue rearrangements and early developmental cues [33]. Most of the germ-layer specifying genes—*GATAb* and *Otx* (ectoderm); *Goosecoid*, *Brachyury*, and *Tbx2/3* (mesoderm); and *Fox-A1* and *GATAa* (endoderm)—are expressed during both asexual reproduction (palleal budding) and whole-body regeneration (vascular budding) [34].

The co-option of patterning genes (or gene modules) during organogenesis explains only in part the morphological similarities between zooids produced by embryogenesis and blastogenesis. Based on the expression dynamics of *Tbx1/10*, *Ebf*, *Mrf*, and *Myh3* for the body wall and *FoxF*, *Tbx1/10*, *Nk4*, and *Myh2* for heart development, Maria M. Prünster et al. [35] show that only a subset of the myogenic transcription factors engaged during ascidian embryogenesis are also involved during blastogenesis.

The partial co-option of developmental modules has also been shown in neurogenesis, but this happens in a somehow circuitous way. During the metamorphosis of solitary ascidians, part of the larval nervous system is recruited to form the adult central nervous system (CNS) through neural stem-like cells (ependymal cells). The antero-posterior (AP) gene expression patterning of the larval CNS regionalizes the distribution of the ependymal cells, which embodies the positional information for the neurons of the adult nervous system. In colonial ascidians, the CNS of asexually developed zooids has the same morphology as the CNS of the post-metamorphic zooids, but it develops in a completely different way [35]. In each blastogenic cycle, neurogenesis starts de novo from a neurogenic transitory structure, the dorsal tube. The dorsal tube combines a role in neurogenesis with a function as a provider of positional clues for neuron patterning, equivalent to the role of the larval CNS markers [35]: in blastogenesis, the dorsal tube could represent a provisional scaffold for AP patterning of the adult CNS, just like the larval CNS does during sexual development [35].

Again, in *Botryllus schlosseri*, the general molecular profiles of embryogenesis vs. blastogenesis are largely distinct despite the shared body plan, organs, and tissues that develop. However, the relative timing of organogenesis and the ordering of tissue-specific gene expression are conserved [36]. Although embryogenesis and blastogenesis can be considered transcriptionally distinct, there are two blocks of stages with shared expression: (i) an earlier block, corresponding to the transition between morula and the early embryogenesis, shows transcriptomic profiles similar to those between the transition from the secondary to primary bud and the stage of secondary bud in blastogenesis; and (ii) a second block, in which the transcription patterns typical of the adult zooids originated by blastogenesis unfold during embryonic development in the temporal segment from mid wrap to oozooid [36].

There is a significant similarity between the embryonic expression of developmental genes in amphioxus and zebrafish, and the corresponding expression during blastogenesis in *B. schlosseri*, but not with embryonic expression in the latter [37]. The differences found in *B. schlosseri* during embryogenesis are likely due to the metamorphosis, a process that zebrafish and amphioxus do not undertake [37].

## 4. Adaptational and Teleological Views

Some degree of teleology is intrinsic to the adultocentric view in which GRN analysis is usually framed. For instance, according to Yen-Chung Chen and Claude Desplan [5] (p. 92, italics added), in the development of the visual system of *Drosophila*, “five subnetworks are … wired differently to fit the specific needs of each region”, for example, a “[p]rogenitor expansion subnetwork [that] makes sure the tissue is formed in the proper size”.

Another case in point is provided by a developmental property of type I developing invertebrates that has often been interpreted in a strong adaptationist sense [30]. This interpretation, in relation to which Eric H. Davidson first applied a description in terms of GRNs [8], is already evident in the term “set-aside cells” designating the cells from which the adult body will develop [38]. The set-aside cells’ property of suddenly activating mitosis and differentiation, independent from the remaining (larval) cells, could be a way to go quickly over metamorphosis, a phase when the animal is particularly exposed to predation and other risks [39]. It would be preferable to say that those cells, rather than being set aside for further development, were temporarily excluded from the current path of ontogenesis. In Richard J. Bird’s words, “it is possible to attribute the existence of any feature to selective advantage [only] if this is the idea you start with” [40] (p. 154).

## 5. The Development of Unicells and Its Regulation

According to a traditional perspective, “to study development is to study multicellularity” [41]. However, the exclusion of unicells from developmental biology is unwarranted [42]. Along their life cycles, several unicells undergo dramatic and predictable transitions, besides those corresponding to progression along the mitotic cycle. An example is trypanosomes [43].

The different developmental stages of trypanosomes, characterized by presence vs. absence, position, and the length of the flagellum, are not separated by cell division. Indeed, we could describe the transition from the amastigote (without flagellum) to the epimastigote (with flagellum) condition as a process of morphological differentiation. In *Trypanosoma*, the temporal regulation of the developmental progression along the cell cycle is accompanied by gene expression changes, mediated almost exclusively at the post-transcriptional level [44]. Trypanosomatids utilize polycistronic transcription to produce most protein-coding mRNAs [45].

Another case in which unicells undergo a dramatic and predictable transition is the conversion between the stage of the cyst (infectively inert but essential for transmission) and the trophozoite (responsible for the invasion of the host tissue) in the amoeba *Entamoeba histolytica*. This change involves the developmental regulation of ca. 15% of the annotated genes [46].

Changes in transcriptome profiles along the life cycle of unicells are comparable to those occurring along the development of metazoans. For example, in *Plasmodium falciparum*, over 80% of the 5409 predicted open reading frames revealed changes in transcript abundance during the maturation of the parasite within the red blood cells of the host. Transcriptional changes during the asexual intraerythrocytic cycle [47] quantitatively resemble those of the early developmental stages in *Drosophila melanogaster*, where 88% of the developmentally modulated genes are expressed during the first 20 h of development [48]. The following examples from the two unicellular eukaryote lineages closest to metazoans may help in bridging the gap between unicell and multicellular development.

*Capsaspora owczarzaki* is a parasitic unicell that attacks the sporocysts of the flatworm *Schistosoma mansoni*, which in turn is a parasite of the freshwater snail *Biomphalaria glabrata* [49,50]. *Capsaspora* differentiates into three temporally distinct cell types: the proliferativetrophic stage (a filopodiate amoeba); an aggregative multicellular stage; and a cystic resistance form [51]. Temporal cell-type differentiation during the life cycle of *Capsaspora* is finely regulated at the level of protein abundance and phosphorylation, like different animal cell types and tissues [52].

Choanoflagellates are free-living unicells whose affinities to animals have been suggested based on their morphological similarity to a cell-type characteristic of sponges (the choanocytes) and because several choanoflagellate species alternate between solitary and multicellular (colonial) stages. A complex life cycle has been described in *Salpingoeca rosetta*, one of these colony-forming choanoflagellates, with changes between different cell types during both unicellular and colonial phases [53,54]. It may be sensible to distinguish between temporal cell types, characterizing different developmental phases, and spatial cell types, coexisting in the colonial or multicellular organism at the same ontogenetic period [55]. Still, as suggested in the last section of this article, this distinction should not be overemphasized.

## 6. Structural and Functional Hierarchies

### 6.1. The Traditional View

In addition to the concept of development as the progressive sequence of changes from egg to adult, the traditional perspective on GRNs takes for granted a hierarchical nature of body structure and development alike [56]. In 1878, Carl Gegenbaur summarized this view of development in the following terms:

These processes of differentiation consist in the more or less similar morphological elements (cells) which represent the organism, acquiring, in larger or smaller groups, distinct characters: in their being differentiated, and forming the rudiments (first stages) of organs, by taking a definite order and arrangement. These organs then are made up of cells, which form their tissues. We thus arrive at the essence of the architecture of organisms; we have tissues, which make up organs, and are themselves composed of form-elements—the cells.

[57] (p. 20)

The hypothesis of a hierarchical organization of the animal seems to require a causal explanation through equally hierarchical mechanisms [58]. The logical steps of this argument are summarized in the following excerpts:

Development of animal body plans proceeds by the progressive installation of transcriptional regulatory states, transiently positioned in embryonic space. The underlying mechanism is the localized expression of genes encoding sequence-specific transcription factors at specific times and places.

[59] (p. 4835)

Developmental programs comprise the sets of stepwise changes in cells, tissues, and organs that ultimately produce phenotypes. The developmental program of a given phenotype is generally controlled by one or more GRNs.

[7] (p. 2)

The GRNs controlling embryonic development of the body plan are intrinsically hierarchical, essentially because of the number of successive spatial regulatory states that must be installed in the course of pattern formation, cell-type specification, and differentiation. If the regulatory state defines a progenitor field for a given organ, then all the subsequent stages in the development of that organ must take place within that domain.

[24] (p. 973)

However, this hierarchical organization of mechanisms is not a description of GRNs; it is instead a kind of generative rulebook they must obey. This a priori conceived reading fits into the traditional metaphor of the morphogenetic tree [60]. In the words of Rudolf Raff, who aptly criticized its legitimacy and heuristic value:

The morphogenetic tree is a diagrammatic construct that represents ontogeny as a tree, with causal connections between hierarchical levels represented as the branches. … This model is extremely simple and understandable, and it allows predictions to be made. But heuristic elegance comes at a high cost. To achieve simplicity, the nature of developmental processes is ignored, and some very static (and demonstrably incorrect) assumptions about development have to be built in. The most serious explicit assumption is that genes acting early in development have larger effects on adult phenotype than those acting later. Such a view … is a gross oversimplification, and it leads to a number of misleading predictions about development and how development must evolve.

[61] (p. 518)

### 6.2. I “Know” What Needs to Be Done, So There Has to Be a Mechanism to Do It

If a hierarchical organization of body features is taken for granted, we may be tempted to explain it as the product of a similarly hierarchical system of developmental modules. In turn, hierarchical regulatory modules will be hypothesized to be responsible for the emergence of largely conserved developmental modules. The evolution of this system will thus be due to the changes in individual regulatory modules or individual links in their overall wiring.

**Figure 1:**
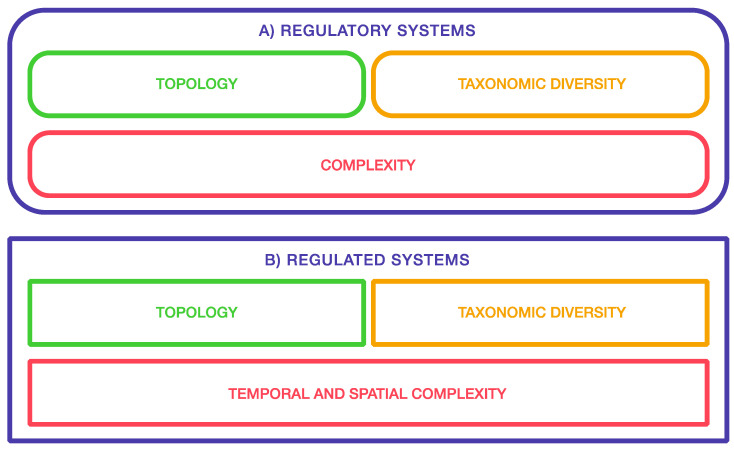
Both regulatory systems (**A**) and regulated systems (**B**) are complex and diverse. However, to assume a precise matching among them is unwarranted. To examine the extent and the topology of this mapping, we must first develop independent pictures of the structural and functional properties of GRNs on one side and those of organisms on the other side.

Unfortunately, this argument does not provide proof of a hierarchical regulatory organization. There is no a priori reason to assume one-to-one relationships between phenotypic features (especially those corresponding to major units such as individual organs or body parts) and the regulatory modules controlling their production. The complexity of the genotype to phenotype mapping [62] requires independent assessment of the not-necessarily hierarchical structure of the regulatory machinery of development on one side and of the architecture of the organism on the other (Figure 1).

This does not rule out the possible existence of Character Identity Mechanisms (ChIMs), intended as “cohesive units with a recognizable mechanistic architecture that is traceable through evolution even though this architecture can be multiply realized and exhibit diverse etiological organization”. [63] (p. 2). However, it would be premature to hypothesize a hierarchical organization of these ChIMs before we know enough about their composition, distribution, and diversity.

James DiFrisco, Alan C. Love, and Günter P. Wagner describe ChIMs as conserved mechanisms that have a distinctive biological role in trait individuation. Under this definition, ChIMs remain on a level of abstraction that can accommodate a variety of molecular and cellular mechanisms contributing to character identity (i.e., differently constituted mechanisms with diverse etiological organization) [63]. Following their account, “the most direct experimental evidence for the existence of ChIMs comes from the recognition that the role of the Hox gene *Ultrabithorax* is to control character identity rather than phenotype” [63] (p. 3); cf. [64,65]. Experimental knockdown of *Ultrabithorax* in the beetle *Tribolium* led to the development of elytra (character identity of beetle forewings) on the metathoracic segment (positionally, the site of the hindwings), indicating separate control of position vs. character identity. Remarkably, this example would not support a model of hierarchical *organization* of the regulatory system but a combinatorial one [66].

### 6.3. From Single-Cell Sequencing to Modeling the Topology of Developmental Systems

Single-cell gene expression is inherently stochastic [67], a property increasingly incorporated in algorithms for inferring cell-state transitions, e.g., [68]. Single-cell RNA sequencing, now a standard technique used to characterize development at the tissue level, has revolutionized the study of evolutionary developmental dynamics [69,70,71]. One example of the usefulness of this increasingly popular technique is the assessment of lineage differentiation trajectories [72,73]. In such a dynamic, GRNs are viewed as regulating the probability of cells to move toward stable “attractor” states, possibly corresponding to the observed cell types [72]. This network analysis has also been applied to the study of tumors, e.g., [74]

The efficiency of most lineage reconstruction methods depends on limiting the search to a particular lineage graph topology [75]. This potentially entails serious heuristic biases, as most methods are designed to only find chains and trees [76]. Implicitly (and often also explicitly), lineage reconstruction methods are generally framed in terms of Conrad Hal Waddington’s epigenetic landscape [77], a metaphor used to represent the relationship between gene expression and cell-type specification during development [68,69,70,75,78,79,80,81,82,83].

However, the application of this framework may skew the resulting tree-like topology. This bias has possibly historical roots [42] in the tree-like topology map used to describe the cell lineage of *Caenorhabditis elegans*, the first ever constructed [84]. Hence, algorithms used to infer lineage maps are biased toward the production of tree-like and chain-like topologies [85].

To circumvent this systematic error, Somya Mani and Tsvi Tlusty [86] have developed a model that generates multiple differentiation pathways deployed during development without imposing a predefined branching topology. Thanks to this method, Mani and Tlusty analyze the topologies of the resulting differentiation trajectories of the organism’s cell types a posteriori. In their simulations, five qualitatively different topologies were recovered, one of which comprises acyclic graphs with branches (thereby tree-like); the others being unicellular graphs, cyclic multicellular graphs, chains (i.e., acyclic graphs with no branches), and acyclic graphs with both branches and links connecting branches (thus making them nontree-like). Remarkably, tree-type lineage differentiation maps are the rarest in Mani and Tlusty’s set. This would imply that cell-type lineage graphs are unlikely to be tree-like [86]. Instead, the most common would be acyclic graphs where multiple developmental routes end up at the same cell type [42]. Examples of nontree-like lineage maps have been reported for zebrafish development [87] and hydra adult homeostasis [88]. Based upon these and other examples, it may be expected that different topologies coexist within one and the same cell-type lineage graph.

However, we must abstain from generalizing considering only a bunch of examples. The topology of cell-type lineages may be very different across metazoans, perhaps even while comparing closely related taxa, especially if the topology is per se a target of natural selection. This possibility is suggested by an intriguing correlation emerging between cell-lineage topology and the capability for whole-body regeneration [86]. Indeed, the presence of adult pluripotent cells would be a prerequisite for whole-body regeneration. In turn, pluripotent cells are likely to be a part of the adult organism, as suggested by simulations where cells potentially allow whole-body regeneration to show up in most acyclic lineage graphs (73.3% of all graphs) [86].

Among metazoans, in agreement with a hypothetical scenario of developmental inertia, whole-body regeneration is likely to be an ancestral trait that might precede the evolution of increasingly fine regulatory systems [89]. The limited capacity for regeneration observed in various animal clades thus represents multiple instances of loss of this capacity, itself a trend open to explanations in terms of adaptation [90]. If this condition is tightly coupled with cell-lineage topology, selection in favor of reduced regeneration capacity would translate into selection in favor of cell-type lineages that do not lead to the production of adult pluripotent cells but to lineages with branching topology instead. This scenario may easily suggest the framework for experimental comparative tests.

## 7. Spatial and Temporal Dimensions

### 7.1. Time and Space, Intertwined

In the processes of developmental regulation, distinguishing between temporal and spatial dimensions is often difficult:

A distinction between temporal and spatial aspects of the molecular control of development is often artificial. This is true, in particular, in the context of the collinearity between the spatial organization of the Hox genes along the chromosome, the temporal sequence of their activation and the spatial order of the regions along the animal’s main body axis, in which each of these genes is expressed. Therefore, it is wise to identify a search for correspondence between spatial (morphological) and temporal (developmental) units and patterns as a primary target of developmental (and evo-devo) biology.

[23], pp. 55–56; on collinearity, cf. [91,92,93]

In *Drosophila*, differentiation and patterning of specific structures are the result of a combinatorial play of sex-, tissue- and stage-specific gene expression [94,95,96,97,98]. For example, the generation of neural cell diversity depends on a temporal and spatial patterning of neural progenitors [5,99]. In flies belonging to different strains, up to 50% of the expressed genes have been found at significantly different levels [100,101]. Even a very small and structurally simple organism, such as the multicellular mold *Aspergillus nidulans*, contains multiple differentiated cell types with both spatial and temporal regulation of gene expression [102].

### 7.2. Trade-Off between Spatial and Temporal Differentiation

During evolution, switching from temporal to spatial control of differentiation is arguably more common than usually appreciated. High complexity in the adult is not necessarily coupled with highly complex life cycles with dramatic differences between stages, and vice versa. In insects, no gross increase in the complexity of adult organization evolved in close parallelism with the evolution of holometaboly or hypermetaboly [103]. Within metazoans at large, groups with an unusually complex life cycle (sometimes accompanied by switch points between alternate developmental routes) are often those with simplified adult structures. Examples of this can be found in dicyemids [104], cycliophorans [105], and parasitic flatworms.

In Neodermata (the large group of parasitic flatworms that includes monogeneans, digeneans, and cestodes), the evolution of adult morphology has been extensively simplified in comparison to many free-living groups, including *Bothrioplana*, their putative sister taxon [106]. However, the life cycle of neodermatans has generally become more complex, with the evolution of highly diversified larval stages and alternating generations. This is supported by several transcriptomic studies in digeneans and cestodes (unfortunately, the most comprehensively studied monogenean has transcriptomic data available only for the adult but not for the larval stages) [107].

In the digenean *Opisthorchis felineus*, adult and metacercarial stages seem to have 648 and 903 stage-specific genes, respectively [108]. In the cestode *Hymenolepis microstoma*, differences at the post-transcriptional/translational level have been identified between the larva and the adult worm [109]. Regional differences have been also found in the adult, specifically between the scolex plus neck, mature strobila, and gravid strobila, with differential expression in 4.5–30% of the genes [109].

More impressive contrasts have been found in the cestode *Taenia multiceps*, where the total number of genes differentially expressed in different life stages, from the onchosphere larva to the adult, range between 2577 and 3879. These numbers are much higher than the 1229 to 1939 genes differentially expressed in the different tissues of the adult worm [110].

In another cestode, *Echinococcus granulosus*, 963 genes and 31 microRNAs (miRNAs) are differentially expressed during the transition from protoscolex to adult worm, whereas 972 genes and 27 miRNAs are differentially expressed in the early development of protoscoleces to form cysts [111].

The aforementioned study is an example of a growing interest in the contribution of miRNAs to the emergence of spatial and temporal heterogeneity in developing animals. Whole tissue- and cell-type-specific miRNAomes have been characterized for model species, such as the zebrafish *Danio rerio*, the fruit fly *Drosophila melanogaster*, and the nematode *Caenorhabditis elegans* [112]. In *Danio rerio*, miRNAs play important roles, e.g., as mediators of the maternal-to-zygotic transition and regulators of cell fate specification during early embryogenesis. miRNAs continue to modulate proliferation and differentiation in the neurectoderm, mesoderm, and endoderm during and after gastrulation [113]. In *D. melanogaster*, miRNAs modulate cell proliferation and differentiation in the eye and wing imaginal discs, neurons, germ line, glia, and salivary glands [114]. In *C. elegans*, miRNAs regulate differentiation in the hypodermis, vulval precursor cells, neurons, and germ line and are involved in regulating the formation of the dauer stage [115,116,117,118].

## 8. Concluding Remarks

### 8.1. GRNs and Phenotypes

Conceptual issues troubling current explanations of developmental processes and morphological characters in terms of GRNs have been actively discussed by recent papers, e.g., [119,120]. One of the problems debated by James DiFrisco and Johannes Jaeger [120] is the disregard of the fact that phenotypic evolution is to a large extent dissociable from the evolution of GRNs (even strongly conserved characters may result from different developmental processes in different lineages). This “network drift” or “developmental system drift,” caused by mutations and polymorphisms in regulatory network interactions, does not seem to affect the outcome of a developmental process [121,122].

It is therefore questionable to look for Organogenetic Gene Networks [123] as units of genetic control of organ formation. Any organ of a certain complexity is the outcome of many developmental processes, only some of which are likely to be specific to the organ. Moreover, organs are at the same time morphological units distinguishable by the specific structure and position within the body and/or functional units [124], but we cannot assume that they are also (macro)modules from a morphogenetic point of view.

### 8.2. No Universal Regulatory Metaphor

The popular models of GRNs are strongly rooted in the developmental biology of certain zoological groups (e.g., sea urchins). However, only from a perspective open to all biological systems can we accept that “development is a system property of the regulatory genome” [56] (p. 35). As discussed above, gene regulatory logics, whatever their intrinsic topology, also apply to the development of unicells no less than to the control of cellular differentiation and form across metazoans. Even if we restrict focus to multicellular organisms, in these investigations, we should also include plants, the most conspicuous group of non-metazoan multicellular organisms. It is not surprising to find GRNs mentioned in papers on plant development [125], regeneration [126], or evolution [127,128]. However, the GRNs of plants may not work in the same way as those of the sea urchin. While using the same language in describing the regulation of development in all branches of the tree of life, we are moving toward a level of generalization that must be moderated by repeatedly checking the actual significance of this metaphor, unless we prefer mathematical abstractions to molecular explanations. This step would require abandoning the projections of GRNs toward any hypothetical hierarchical model.

### 8.3. Spatially and Temporally Distributed Complexity

Evolution is not limited to changes “in place”, i.e., changes in a GRN within a common (homologous) tissue between species [129]. This myopic scenario is equivalent to that part of the race between Achilles and the tortoise in which the swift-footed Achilles approaches the slow-moving reptile without ever reaching it. However, in the real world, it is only a matter of time before Achilles overcomes the tortoise. Similarly, evolution can rapidly extend beyond local replacement of a cell type with a different cell type or displace the boundary between “territories” under the control of separate GRNs. A further step is variation (increase or decrease) in the structural complexity of the animal; another is a change in life-cycle complexity. Eventually, the trade-off between spatial and temporal aspects of complexity is arguably more frequent than expected and with more conspicuous evolutionary consequences. A first suggestion in this direction was Aleksei Alekseevich Zakhvatkin’s [130] Synzoospore hypothesis.

Zakhvatkin [130] proposed that the last common ancestor of the Metazoa might have been an organism that already exhibited different cell types during different life-history phases (temporal cell disparity and cell differentiation), which also happens in many extant unicells (see Section 5 above). This means that a first degree of cell differentiation within a multicellular organism may have originated by the stabilization and increased predictability of a pattern of coexistence of alternative cell phenotypes [51,131,132,133,134,135]. If multicellularity sets the most obvious context for cell and tissue differentiation, it is also likely that cell differentiation might have led to multicellularity [136]. In Kirill V. Mikhailov et al.’s further elaboration of Zakhvatkin’s hypothesis [131], metazoan multicellularity resulted from the consistent combination of different stages in a multiphasic choanoflagellate life cycle.

The essence of Zakhvatkin’s hypothesis is that the internalization of cues inducing the expression of what originally were alternative phenotypes of a polyphenic unicell may have given rise to the orderly and predictable coexistence of these phenotypes in a precise spatial array. In some lineages, history may have also run the other way. Alessandro Minelli and Giuseppe Fusco [18] have suggested that a temporally (rather than spatially) consistent, predictable sequence of phenotypes may have evolved from a polyphenism, giving rise to structurally distinct stages within a complex life cycle.

Ultimately, our critical perspective on GRNs acknowledges the importance of this inference method to describe the molecular mechanisms involved in the development and differentiation of metazoans, and it highlights the importance of a multifactorial approach that incorporates the taxonomic diversity and the spatiotemporal complexity of all eukaryotes.
